# Real supermodels wear wool: summarizing the impact of the pregnant sheep as an animal model for adaptive fetal programming

**DOI:** 10.1093/af/vfz018

**Published:** 2019-06-25

**Authors:** Kristin A Beede, Sean W Limesand, Jessica L Petersen, Dustin T Yates

**Affiliations:** 1 Department of Animal Science, University of Nebraska – Lincoln, Lincoln, NE; 2 School of Animal and Comparative Biomedical Sciences, The University of Arizona, Tucson, AZ

**Keywords:** developmental origins, DOHaD, fetal stress, intrauterine growth restriction, placental insufficiency

**Implications**

- Intrauterine growth restriction (IUGR) continues to be a global epidemic that is associated with high early-life mortality rates and greater risk for developing metabolic disorders that lower length and quality of life in affected individuals.
- Fetal programming of muscle growth and metabolic function associated with IUGR is often comparable among nonlitter bearing mammalian species, which allows much of the information learned in domestic animal models to be applicable to humans (and other animals).
- Recent studies in sheep models of IUGR have begun to uncover the molecular mechanisms linking adaptive fetal programming and metabolic dysfunction.
- Targets of adaptive fetal programming indicated by sheep studies include adrenergic and inflammatory pathways that regulate skeletal muscle growth and glucose metabolism. Adaptive changes in these pathways represent potential focus areas for prenatal interventions or postnatal treatments to improve outcomes in IUGR-born offspring.

## Introduction

Low birthweight due to intrauterine growth restriction (IUGR) encumbers United States and global livestock production by increasing neonatal mortality, reducing growth efficiency, and diminishing carcass yield and quality. Low birthweight food animals that survive typically exhibit impaired muscle growth and metabolic dysfunction that leads to poor feed conversion, lighter carcasses, and reduced carcass merit (reviewed by Yates et al., 2018). The growth and metabolic pathologies associated with low birthweight are among the top One-Health, or multi-species, concerns. In addition to livestock, metabolic dysfunction has been observed in low birthweight dogs, horses, wildlife, and humans (reviewed by Yates et al., 2018). In fact, the consistency with which characteristics of IUGR are shared among mammals makes information ascertained from research in livestock highly translatable to humans. Even as toddlers, low birthweight children can exhibit insulin resistance, higher blood pressure, and greater body fat that progresses to diabetes, hypertension, and obesity in adulthood as detailed in previous reviews (Anthony et al., 2003; Limesand et al., 2018; Yates et al., 2018). A fundamental step in improving health outcomes of low birthweight individuals is to identify and target the molecular mechanisms underlying stress-induced fetal programming of poor growth and metabolism. Until recently, very few advancements had been made in this area and the mechanisms driving IUGR pathologies were largely unknown. In the last decade, however, several research groups across the globe have prioritized building a better understanding of IUGR fetal programming from a mechanistic perspective. These efforts have been aided by powerful sheep models that recapitulate the low birthweight conditions and outcomes reported in both humans and other animal models (Anthony et al., 2003; Yates et al., 2018). The objective of this review was to present an overview of these sheep models of IUGR and to discuss how they provide a fundamental basis for further improvement of low birthweight outcomes. As part of this discussion, we will highlight the integral role that several fetal and neonatal sheep models of IUGR have played in narrowing this gap in knowledge.

## Sheep Models for Recapitulating Characteristics of the IUGR Human Fetus

**Figure 1.**
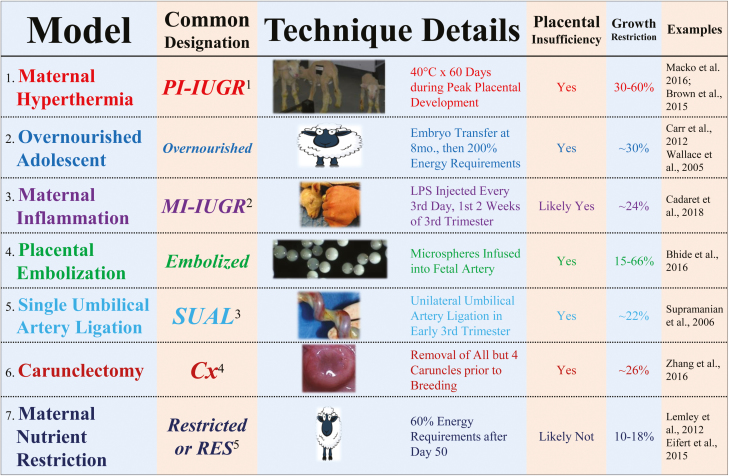
Sheep models that recapitulate the pathologies of IUGR humans. ^1^Placental insufficiency-induced intrauterine growth restriction (PI-IUGR). ^2^Maternal inflammation-induced intrauterine growth restriction (MI-IUGR). ^3^Single umbilical artery ligation (SUAL). ^4^Carunclectomy (Cx). ^5^Nutrient-restricted (RES).

The pregnant sheep is the ideal model for studying human-applicable intrauterine conditions, fetal development, and neonatal outcomes for several reasons. Most importantly, the relative developmental milestones of the sheep fetus are similar to those of the human fetus, and their propensity for singleton or twin pregnancies avoids the confounding factors of litter bearing in rodents and pigs. In addition, the sheep fetus is tolerant of surgical and experimental manipulation due to high placental progesterone production in late gestation, and their large size allows for greater volume and frequency of blood/tissue sampling (Anthony et al., 2003). Several experimental techniques have been used to create IUGR in sheep. Most of these rely on inducing placental insufficiency by either stunting placental development or mechanically diminishing placental structure. Some common techniques for either producing or mimicking placental insufficiency in the pregnant ewe are described below and summarized in Figure 1.

### Maternal hyperthermia

Hyperthermic induction of IUGR in pregnant ewes is a well-characterized sheep model of IUGR (Hay et al., 2016; Limesand et al., 2018). Maternal heat stress reliably produces placental insufficiency, thus recreating the most common cause of IUGR in humans and animals. Regular experimental use of the model began in the late 1980s following earlier proof-of-concept studies in which heat-stressed Merino ewes produced smaller placentae and lambs as described in our recent review (Yates et al., 2018). Over time, the parameters for this model have been refined to 35–40 °C and ~35% relative humidity for 50 to 60 consecutive days beginning around the 40th day of gestation (Hay et al., 2016; Limesand et al., 2018), although the degree of growth restriction can be reduced with fewer days in heat (Galan et al., 1999). The hyperthermic timeframe corresponds to peak placental growth, and thus fetal growth restriction results from placental insufficiency and not fetal hyperthermia per se. Because of this, fetal growth restriction and the associated pathologies are not present at the end of the hyperthermic period (day 90 to 105), but appear soon after and progressively worsen as the fetus outgrows the stunted placenta (Limesand et al., 2018). This model of placental insufficiency-induced intrauterine growth restriction, or PI-IUGR, consistently reduces placental mass by up to 64% and fetal growth by 30% to 60% at term (Anthony et al., 2003; Limesand et al., 2018). Although highly regarded and well characterized, the PI-IUGR model requires the availability of environment-controlled chambers large enough for sheep, which can be a limitation.

### Overnutrition in young ewes

Another technique for producing fetal growth restriction via placental insufficiency is to overfeed adolescent ewes for the majority of gestation, but particularly during the second and third trimesters. For this model, which is utilized by the research group of J.M. Wallace among others, young ewes are implanted with embryos just after puberty and then fed diets containing ~200% of their normal energy requirements (Carr et al., 2012). In a recent review, Wallace (2019) described reductions in placental size of ~40% and asymmetric fetal growth restriction of ~30%, which is comparable to the hyperthermic PI-IUGR model. The paradoxical reduction in placental growth appears to be due to a disruption in endocrine regulation of the nutrient partitioning between maternal and conceptus tissues, which is further compounded by the fact that the dam herself is still growing. Changes in maternal endocrine status include increases in prolactin, insulin, IGF-I, thyroid hormone, and leptin, as well as decreases in progesterone, placental lactogen, and estrogen (Wallace, 2019). The similarities between fetal pathophysiologies in this and other animal models are beneficial attributes of this model. However, the technical skill necessary for embryo transfer is a limitation of the model. In mature ewes, there are stark inconsistencies regarding whether overnutrition leads to fetal growth restriction or fetal overgrowth (Tong et al., 2009). These inconsistencies make overnutrition a difficult model for IUGR but are important in understanding the complex effects of maternal obesity on fetal programming.

### Maternofetal inflammation

We recently performed a study utilizing maternal inflammation induced by iv injection of bacterial lipopolysaccharide every third day for the first 2 wk of the third trimester (day 100 to 113 of gestation) (Cadaret et al., 2018). The endotoxin protocol produced sustained maternal inflammatory responses characterized by increased circulating leukocytes, a febrile response of 0.3 to 0.5 °C, and an increase in plasma TNF-α concentrations. Fetal catheters were not surgically placed until after the conclusion of the injection protocol, and thus we were not able to measure fetal conditions in response to maternal inflammation. Two weeks after the final injection of endotoxin, however, we observed reduced numbers of total white blood cells, lymphocytes, and monocytes in fetal circulation. When necropsied at 125 days of gestational age, we observed a ~22% reduction in fetal mass associated with maternal inflammation. Although we have not yet assessed placental function or morphology, a ~33% reduction in fetal blood glucose together with maternal euglycemia would indicate reduced placental transport of glucose. Although this model of maternal inflammation-induced IUGR, or MI-IUGR, produced milder fetal growth restriction than the hyperthermic PI-IUGR model, we found similar metabolic dysfunction and impaired muscle growth, as described in later sections. It should be noted that this model may be reflective of the chronic inflammatory conditions of maternal obesity (Yan et al., 2011a, 2011b; Ghnenis et al., 2017) and overeating (Jones et al., 2018), which produced both maternofetal inflammation and postnatal metabolic dysfunction.

### Microsphere embolization, umbilical artery ligation, and carunclectomy

The above models create placental stunting indirectly by imposing maternal conditions that disrupt uteroplacental blood flow during placental development. However, techniques described in this section impair placental function by directly diminishing structural components of the placenta. In one model, 150-μm microspheres are embedded into placental capillaries by infusing them into the surgically catheterized descending aorta of the fetus (Bhide et al., 2016). This technique causes an almost 40% increase in placental vascular resistance and reduces placental blood flow by 33% (Bhide et al., 2016). The reduction in placental and fetal growth after placental embolization is comparable with the PI-IUGR model (Anthony et al., 2003). Surgical procedures to ligate the umbilical artery or to remove endometrial caruncles from the uterus have also been used to reduce placental efficacy. Single umbilical artery ligation, or SUAL, is typically performed early in the third trimester of pregnancy and reduces fetal growth by ~22% (Supramaniam et al., 2006). Endometrial carunclectomy of all but four functional caruncles about 10 wk prior to breeding reduced fetal growth by ~26% near term (Robinson et al., 1979; Zhang et al., 2016). These techniques provide the potential for more precise control over the magnitude of placental restriction than the indirect models, but the required surgical procedures can limit the practicality of all three of these models for many research groups.

### Nutrient restriction

Fetal growth restriction can be achieved through restriction of maternal nutrients, although it is unclear whether it is mediated by placental insufficiency (Anthony et al., 2003). The research groups of K.A. Vonnahme and C.O. Lemley have shown that feeding pregnant ewes at ~60% of their recommended energy level beginning on the 50th day of gestation reduced fetal mass by 10% to 18% at day 130 but had minimal impact on placental size and vascular development (Lemley et al., 2012; Eifert et al., 2015). Moreover, undernutrition does not affect placental transport or fetal concentrations of oxygen, and the fetal growth restriction appears to be more symmetrical (summarized by Anthony et al., 2003). Despite these key differences from other sheep models, it is worth noting that the maternal nutrient model reflects a common etiology for maternal stress in humans and animals. Moreover, the ease with which it can be implemented increases its value as a research model.

## Pathologies Associated with Intrauterine Growth Restriction

### Placental insufficiency creates poor intrauterine conditions

**Figure 2.**
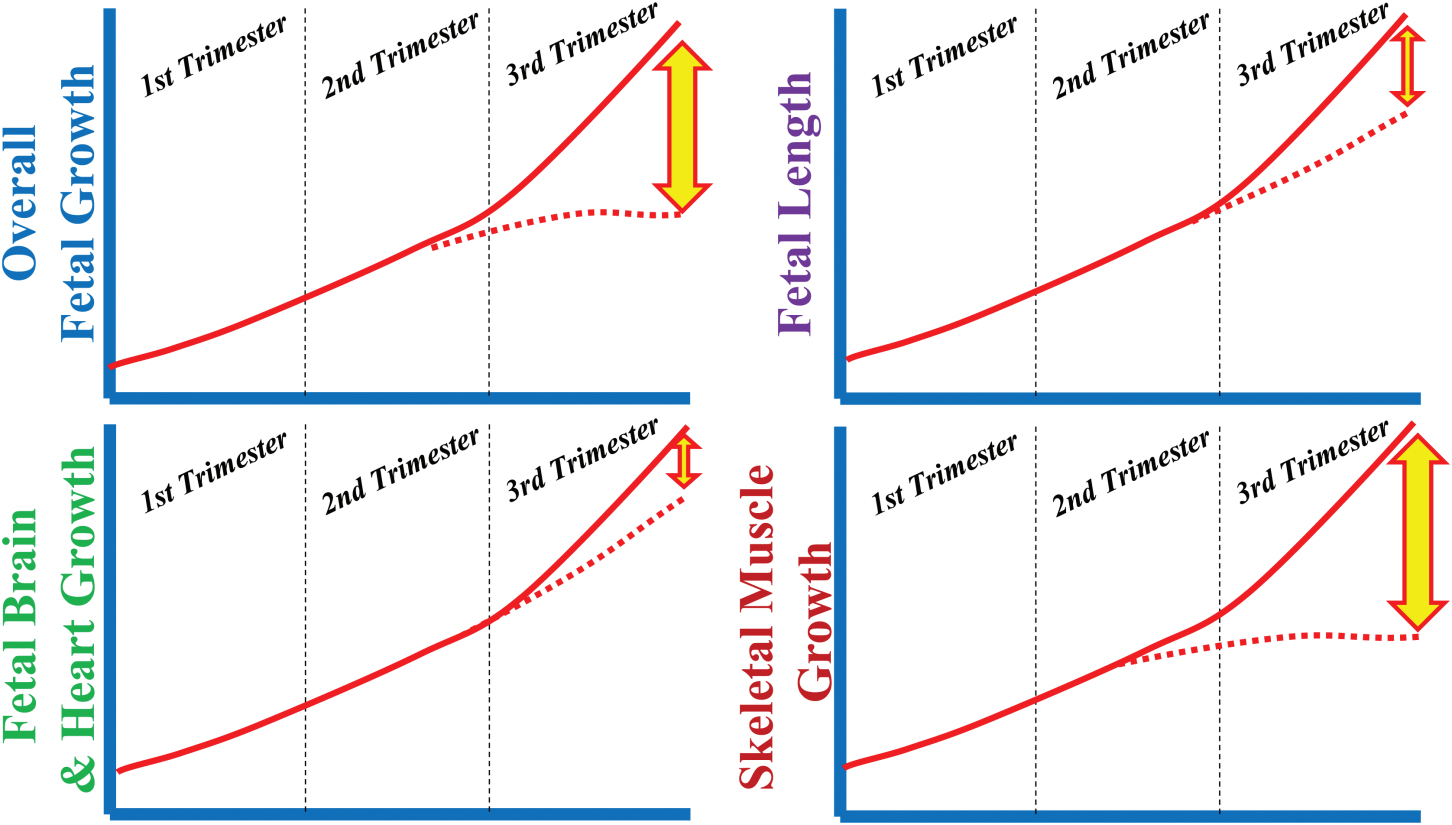
Asymmetry of pathological intrauterine growth restriction. Expected growth rates for uncompromised fetuses and IUGR fetuses are depicted by solid lines and dotted lines, respectively. The differences in the magnitude of growth restriction (yellow arrows) for skeletal muscle compared to structural (length) or vital organ (brain, heart) shows the asymmetry of IUGR. Interpreted from findings reported by Galan et al. (1999).

**Figure 3.**
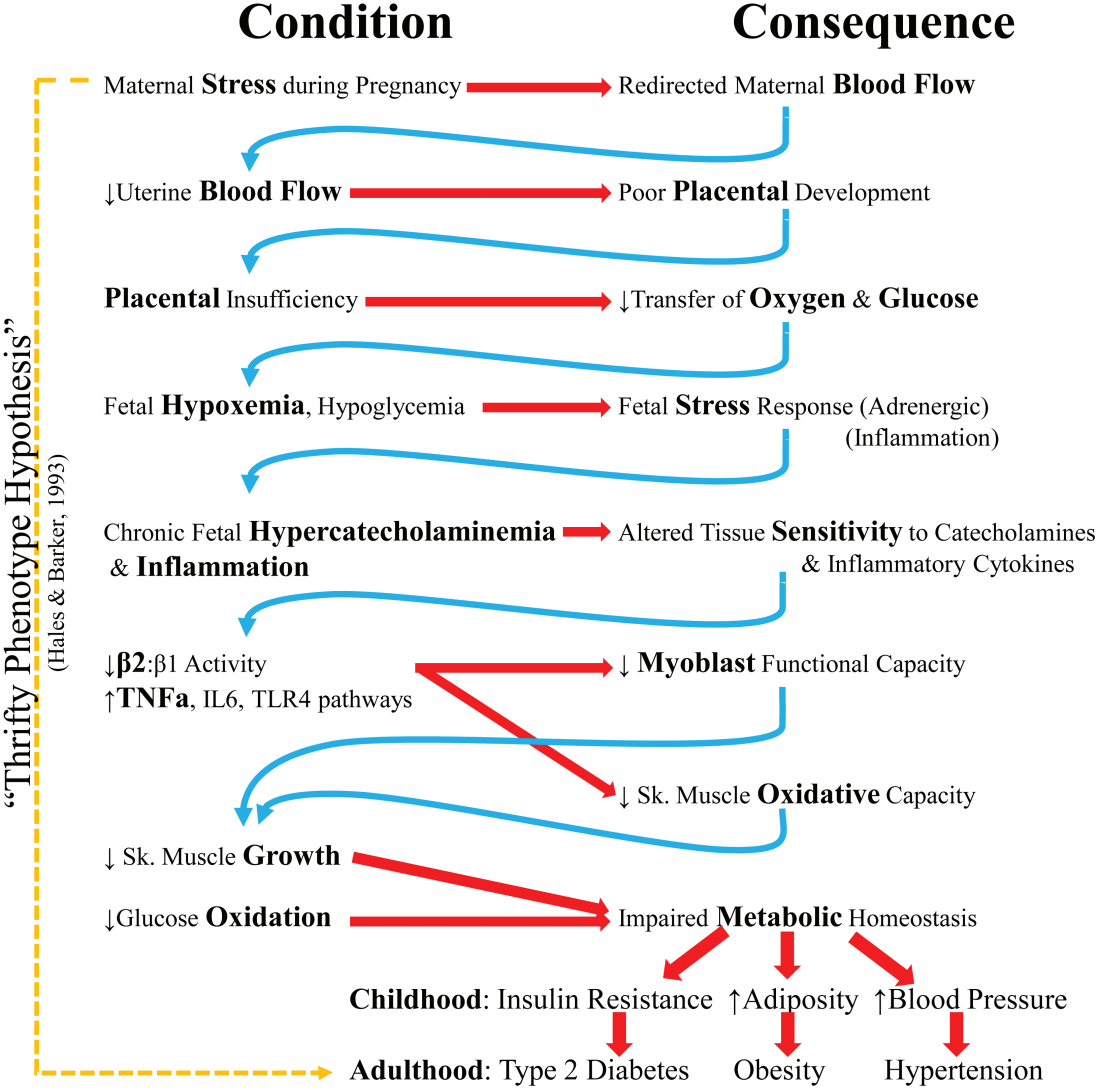
Stepwise conditions and muscle-centric consequences that contribute to IUGR pathologies. See text for citations.

**Figure 4.**
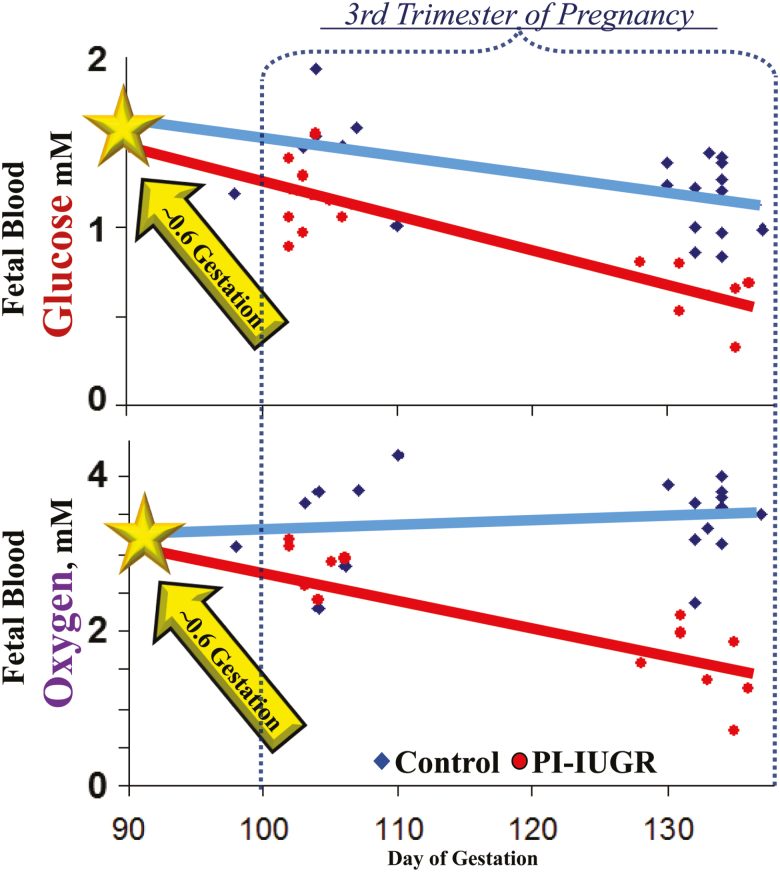
Progressive fetal hypoxemia and hypoglycemia associated with placental insufficiency. These pathologies become apparent in the maternal hyperthermic sheep model of PI-IUGR at approximately 6/10 of the way through gestation, when the fetus begins to outgrow its stunted placenta. These pathologies progressively worsen until birth as the disparity between fetal nutrient requirements and placental capacity to deliver them increases. Based on data included in Macko et al. (2016) and Limesand et al. (2007).

Asymmetric IUGR is associated with postnatal pathologies (Figure 2), many of which involve changes in muscle growth and metabolic capacity (Brown, 2014). IUGR most commonly results from placental insufficiency that limits fetal nutrient and oxygen supply, which sets in motion the series of responses and adaptations summarized in Figure 3. In a previous review, we detailed how impaired placental function and structure lower oxygen and glucose delivery to the fetus, causing fetal hypoxemia and hypoglycemia (Yates et al., 2011). Placental transport of amino acids to the fetus is also reduced, particularly those transported by Systems A (e.g., alanine, serine, proline, glutamine), X_AG_- (e.g., glutamate, aspartate), and y^+^ (e.g., lysine, arginine) (Regnault et al., 2002). A collection of studies led by S.W. Limesand and performed in catheterized PI-IUGR fetal sheep of various gestational ages showed that reduced fetal nutrient and oxygen supplies progressively worsen over the last trimester of pregnancy (Figure 4) as the fetus outgrows its undersized placenta (Macko et al., 2016).

The fetus copes with the disparities in oxygen and nutrient supply in part by mounting the proportional stress responses illustrated in Figure 3 that include amplified circulating noradrenaline and adrenaline (Macko et al., 2016). Using catheterized fetal sheep made hypoxemic via maternal intra-tracheal infusion of nitrogen gas, we found that the spike in fetal catecholamines originates from the *fetal* adrenal gland (Yates et al., 2012). In addition to its role in “fight-or-flight” stress responses, the adrenergic system is a powerful regulator of metabolism and growth (Brown, 2014). High adrenaline concentrations are particularly potent inhibitors of the secretion and action of growth factors, and fetal sheep data show that reduced circulating levels of insulin, IGFs, and other growth-stimulating hormones are concomitant with placental insufficiency (Thorn et al., 2009). In a more direct demonstration, we found that infusion of physiological levels of noradrenaline (the primary fetal adrenal catecholamine) into otherwise healthy fetal sheep near term reduced basal blood insulin by ~61% and completely abolished glucose stimulated insulin secretion (Chen et al., 2017). Furthermore, when we prevented the capacity for rises in catecholamines by surgically ablating the adrenal medulla in fetal sheep, the subsequent impact of both hypoxemia (Yates et al., 2012) and placental insufficiency (Macko et al., 2016) on insulin secretion were diminished markedly. In a study performed by J.L. Morrison’s group, hypoxemia-induced surges in catecholamines redirected blood flow in the sheep fetus toward the vital tissues of the heart, brain, and endocrine organs (Poudel et al., 2015). At the same time, femoral vascular resistance increased and hindlimb blood flow dropped by half (Poudel et al., 2015; Rozance et al., 2018). Re-appropriated blood flow patterns created by hypercatecholaminemia allow the stressed fetus to prioritize nutrient and oxygen delivery to its most vital tissues.

In addition to mounting a chronically-sustained “adrenaline rush,” late-term fetuses also respond to hypoxemia with increased systemic inflammation (Jones et al., 2018). When IUGR was induced in fetal sheep by hypoxemia, circulating levels of the inflammatory cytokines TNFα and IL-6 as well as prostaglandins and activin A were elevated (Bertucci et al., 2011). We also recently found that maternal inflammation at the beginning of the third trimester in led to growth-restricted sheep fetuses whose tissues exhibited evidence of chronic inflammation exposure well after the cessation of maternal inflammation (Cadaret et al., 2018).

### Sustained stress induces adaptive fetal programming aimed nutrient sparing

As the disparity widens between the amount of oxygen and nutrients required for normal growth and the amount supplied by the stunted placenta, fetal tissues undergo developmental adaptations to better match the diminished provisions. Skeletal muscle is a chief target for nutrient-sparring adaptations, as it accounts for more than half of the glucose consumed by the body and upward of 85% of insulin-stimulated glucose utilization (Brown, 2014). The restriction of muscle growth and insulin-sensitive nutrient utilization observed in PI-IUGR fetal sheep (Limesand et al., 2007; Brown et al., 2015; Yates et al., 2016) preserves nutrients for vital heart and brain tissues. In this section, we describe several nutrient sparing adaptations in IUGR fetal sheep indicated by the literature and speculate about some others based on our preliminary findings. Although crucial to fetal survival, these developmental adaptations (illustrated in Figure 2) underlie the poor body composition and metabolic dysfunction that increases health risks in offspring.

#### Reduced muscle mass and altered body composition

Preferential delivery of nutrients to vital organs at the expense of muscle growth results in asymmetric fetal growth restriction that is reflected in altered body composition over the third trimester of pregnancy (Galan et al., 1999; Carr et al., 2012). As neonates, the *bodyweight* of low birthweight offspring often tends to normalize due to postnatal “catch up” growth. However, enhanced growth is achieved by greater than normal rates of fat deposition and not by accelerated muscle growth (De Blasio et al., 2007).

#### Reduced myoblast function

Using a combination of immunohistochemistry and ex vivo functional studies, we have demonstrated that impaired skeletal muscle growth in the PI-IUGR fetal sheep is the product of intrinsic dysfunction of muscle stem cells called myoblasts (Yates et al., 2014, 2016; Posont et al., 2018). Muscle fiber number is static by the early third trimester of pregnancy in most nonlitter bearing mammals. Subsequent muscle growth occurs by hypertrophy, which requires increased fiber nuclei content to facilitate greater protein synthesis. Fibers gain nuclei when myoblasts proliferate, differentiate, and then fuse with existing fibers, effectively donating their nuclei. This is a rate limiting step for hypertrophy, and muscle growth is proportional with myoblast function. When we isolated myoblasts from PI-IUGR fetal sheep at 0.9 of gestation and assessed their function in culture, we found that their capacity for both proliferation and differentiation was intrinsically reduced across a variety of culture conditions (Yates et al., 2014; Posont et al., 2018). The poor performance of PI-IUGR fetal myoblasts compared with control myoblasts cultured under the same conditions shows evidence of *intrinsic* deficits in functional capacity and responsiveness to stimulation, which coincided with impaired myoblast profiles in stained sections of hindlimb muscle and smaller fetal muscle fibers (Yates et al., 2014, 2016).

#### Reduced protein accretion

In concert with its reduced growth, the skeletal muscle of low birthweight offspring utilizes less protein during early growth. A collection of recent studies performed by the laboratory of L.D. Brown described several previously unrecognized changes in protein utilization by IUGR fetal muscle. Using catheterized PI-IUGR fetal sheep, they found that the rate at which skeletal muscle protein is broken down remains comparable to uncompromised fetuses (Rozance et al., 2018). However, substantial reductions in the uptake and utilization of amino acids by PI-IUGR fetal muscle led to corresponding drops of up to 42% in protein synthesis and accretion rates (Rozance et al., 2018). Interestingly, differential changes in both placental amino acid transport systems and fetal utilization rates yielded varying effects on circulating concentrations of individual amino acids (Rozance et al., 2018; Wai et al., 2018). For example, tyrosine, arginine, and isoleucine were reduced in PI-IUGR fetal blood, but taurine, glycine, and alanine were increased (Rozance et al., 2018). Moreover, exogenous amino acids delivered directly to the sheep fetus via infusion failed to improve protein synthesis and accretion rates, muscle growth, and fetal size (Wai et al., 2018). Instead, the extra amino acids were oxidized by the fetus for energy. Thus, reduced protein accretion and muscle growth in the IUGR fetus is not solely due to lower amino acid availability. Rather, less blood flow, oxygen utilization, insulin stimulation, and other factors likely play a combined role along with reduced amino acids in impaired protein accretion in IUGR muscle (Rozance et al., 2018).

#### Greater fat deposition and less fat mobilization

The reduction of skeletal muscle mass and nutrient utilization in low birthweight offspring causes a greater proportion of dietary nutrients to be stored as fat (Wallace et al., 2018). This is facilitated in part by adaptive programming of adipocytes that enhances their ability to both proliferate and grow in size, thus allowing them to accommodate more lipid storage (Desai and Ross, 2011). A potential underlying component of adipocyte programming is greater expression and activity of peroxisome proliferator-activated receptor gamma (PPARγ), particularly in visceral fat of low birthweight offspring (Joss-Moore et al., 2010). PPARγ is a fatty acid-activated nuclear transcription factor that stimulates adipocyte differentiation and uptake of fat from the bloodstream for storage. It is important to note that the reductions in skeletal muscle growth and the increases in fat deposition occur via independent mechanisms. In fact, fat deposits are almost nonexistent in the IUGR sheep fetus, and greater adiposity manifests only when restricted nutrient levels are alleviated after birth.

#### Impaired oxidative metabolism

A pair of studies performed in catheterized PI-IUGR fetal sheep showed that whole-body glucose oxidation rates were reduced despite the fact that glucose utilization rates were normal (Limesand et al., 2007; Brown et al., 2015). Reduced glucose oxidation rates coincided with reduced proportions of oxidative fibers and greater proportions of glycolytic fibers in fetal hindlimb muscles (Yates et al., 2016). We recently found that impaired glucose oxidation rates were primarily due to muscle-centric deficits, as ^14^CO_2_ produced from [^14^C]d-glucose by hindlimb tissues (in vivo) and primary skeletal muscle (ex vivo) was substantially reduced in MI-IUGR fetal sheep (Cadaret et al., 2018). Moreover, our preliminary findings in PI-IUGR lambs at 30 days of age allow us to speculate that muscle-specific metabolic deficits persist in postnatal life. As glucose oxidation wains, some studies indicate that IUGR skeletal muscle utilizes more glucose for lactate production (Limesand et al., 2007; Brown et al., 2015), which would allow muscle to continue clearing glucose while also preserving carbohydrates as lactate. Unlike glucose, lactate can be secreted back into the bloodstream for use in hepatic gluconeogenesis or for energy production by cardiac tissue. We speculate that the shift in glucose metabolism is associated more with hypoxemia and adrenergic responses rather than the low circulating concentrations of glucose or insulin. Most sheep models of IUGR produce substantial fetal hypoglycemia and hypoinsulinemia, which plays a clear role in reduced growth rates. However, acute insulin infusion did not improve oxidative metabolic indices in PI-IUGR fetuses (Brown et al., 2015) and in fact further increased circulating and hepatic lactate concentrations (Jones et al., 2019). Correction of hypoglycemia in the PI-IUGR sheep fetus via endogenous infusion also caused a spike in lactate levels and was in general poorly tolerated by the fetus (Rozance et al., 2009). The impact of IUGR/low birthweight on fat metabolism is substantially less clear. In adult men born with low birthweights, lipid oxidation rates were increased in concert with reduced glucose oxidation rates (Brons et al., 2016). In IUGR-born lambs, however, clearance of triglycerides and free fatty acids from circulation was impaired (Wallace et al., 2014). Amino acid oxidation rates estimated by leucine oxidation were lower in the PI-IUGR fetal sheep (Brown et al., 2012). Interestingly, *acute* infusion of amino acids into PI-IUGR fetuses did not increase leucine oxidation as it did in control fetuses but instead increased protein accretion (Brown et al., 2012). However, when amino acid infusion into PI-IUGR fetal sheep was maintained for 10 d, the increase in protein accretion was diminished and a greater amount of leucine was instead oxidized (Wai et al., 2018).

## Potential Molecular Mechanisms for IUGR Pathologies

### Skeletal muscle regulation by “stress” systems

Adrenergic and inflammatory systems play prevalent roles in stress responses but are also powerful regulators of skeletal muscle growth and function. We observed profound effects of β adrenergic stimulation and inflammatory cytokines on myoblast function and glucose metabolism in sheep (Riley et al., 2016; Barnes et al., 2017; Posont et al., 2018). The regulatory impact of these stress systems on muscle encompasses both direct and indirect effects and can vary depending upon the magnitude and duration of exposure, as described in detail in a recent review (Yates et al., 2018). It is important to note that although the fetal programming mechanisms for IUGR postulated below likely arise in response to chronic stimulation by catecholamines and cytokines, they do not require persistent elevation of these stress mediators after birth. Rather, they represent altered responsiveness of skeletal muscle (and perhaps other tissues) to *normal* concentration fluxes of these regulatory “stress” factors.

### Altered β adrenergic responsiveness in IUGR skeletal muscle

The β2 adrenergic receptor is the predominant isoform in skeletal muscle, although β1 and β3 receptors are also present. However, we found that skeletal muscle and myoblasts from PI-IUGR fetal sheep as well as skeletal muscle from PI-IUGR neonatal lambs express less mRNA for the β2 adrenergic receptor than controls (Yates et al., 2018). Conversely, mRNA expression for the β1 and β3 receptors was not reduced and in the case of fetal myoblasts was even increased. This change in adrenergic receptor profiles is likely an adaptive response to chronic hypercatecholaminemia, as we saw similar gene expression profiles in fetal sheep that were infused with noradrenaline for 7 days. Our ex vivo studies performed in muscle taken from rats, steers, and juvenile sheep showed that β2 adrenergic stimulation increased insulin action and glucose oxidation, but β1 stimulation either had no effect or reduced it (Barnes et al., 2017; Cadaret et al., 2017). Interestingly, β2 receptor mRNA expression in L6 (rat) myoblasts was reduced after 96-hr incubation with adrenaline or inflammatory cytokines (Riley et al., 2016). This presumably contributed to the temporal differences in adrenergic influence on proliferation rates, which were reduced after 4 hr in adrenaline-spiked media but stimulated after 48 and 96 hr. Our preliminary evidence persuades us to speculate that sustained increases in myoblast proliferation rates are not necessarily beneficial. In myoblasts isolated from MI-IUGR fetal sheep, we found that the ~15% greater ex vivo proliferation rates after 72 hr in complete growth media coincided with a ~30% reduction in early differentiating (myogenin^+^) myoblasts and a ~10% reduction in late differentiating (desmin^+^) myoblasts after 72 hr in differentiation media (Beede and Yates, unpublished). The fact that both proliferation and differentiation capacities were reduced in PI-IUGR fetal myoblasts (Posont et al., 2018) shows the impact that varying magnitudes and durations of exposure can have on some IUGR pathologies. Adrenergic adaptations presumably diminish β2-stimulated protein synthesis in IUGR skeletal muscle, which together with intrinsic myoblast dysfunction likely account for much of the programmed impairment of muscle growth capacity. Indeed, when we prevented rises in adrenaline by performing fetal adrenal demedullation at 90 d of gestational age, subsequent growth in PI-IUGR fetuses improved by over 50% (Macko et al., 2016).

### Enhanced inflammatory sensitivity in IUGR skeletal muscle

Like the β2 adrenergic signaling pathway, inflammatory cytokine pathways appear to be altered in muscle and myoblasts of PI-IUGR and MI-IUGR fetal sheep (Yates et al., 2018). Unlike β2 activity, however, inflammatory pathways for TNFα Receptor 1, Interleukin 6 Receptor, and Toll-Like Receptor 4 appear to be *enhanced* by muscle adaptations in MI-IUGR fetal sheep (Yates et al., 2018). In addition, gene expression for TNFα Receptor 1 was greater in PI-IUGR fetal skeletal muscle and expression for both TNFα Receptor 1 and Interleukin 6 Receptor were greater in PI-IUGR fetal myoblasts (Posont et al., 2018). Sustained activity of these NFκB-mediated pathways inhibits glucose oxidation (Liu et al., 2012). Moreover, we have shown that inflammatory cytokines disrupt skeletal muscle insulin signaling (Cadaret et al., 2017). We speculate that their enhanced activity together with reduced β2 adrenergic activity represent the primary mechanistic changes underlying adaptive fetal programming of poor muscle growth and glucose metabolism.

## Conclusions

Intrauterine growth restriction is a leading cause of perinatal mortality worldwide and leaves individuals at 18-fold greater risk for metabolic disorders that reduce length and quality of life. Unlike other major maternofetal pathologies, the prevalence of IUGR in the United States has not fallen over the last two decades. The fetal conditions and postnatal outcomes of IUGR are consistent among most mammalian species, which makes observations in animal models translatable to humans. A number of models developed in pregnant sheep have drastically improved our knowledge of IUGR fetal programming, which provides the fundamental basis for improving health outcomes in IUGR-born individuals.

## About the Authors

**Kristin Beede** is a Research Technologist in the Department of Animal Science at the University of Nebraska – Lincoln. She earned her BS degree in animal science from the University of Nebraska – Lincoln and her MS degree in reproductive physiology at the University of Nebraska – Lincoln. Kristin previously worked as an intern in the research and development group at Schering-Plough Animal Health and as a technician in the veterinary diagnostic group at GeneSeek Neogen Genomics. Kristin’s current research focuses on metabolic fetal programming related to maternal stress, placental insufficiency, and intrauterine growth restriction.

**Sean Limesand** is a Professor of Endocrinology in the School of Animal and Comparative Biomedical Sciences at The University of Arizona. He obtained his PhD in Molecular Endocrinology from Colorado State University and was a Postdoctoral Fellow in Perinatal Biology at the University of Colorado, School of Medicine. His current research programs use an integrative approach at the whole animal, isolated organ, cellular, and molecular levels to determine developmental adaptations in pancreatic β-cells and insulin sensitivity that result from early life risk factors, such as intrauterine growth restriction, and increased risk of glucose intolerance and diabetes in later life.

**Jessica Petersen** is an Assistant Professor of Functional Genomics in the Department of Animal Science at the University of Nebraska – Lincoln. She earned her BS degree in biology from Nebraska Wesleyan University, her MS in biology at Western Illinois University, and her PhD in the Genomic Variation Laboratory at the University of California, Davis. She completed a postdoctoral fellowship in equine genetics and genomics at the University of Minnesota College of Veterinary Medicine. Dr. Petersen’s research program uses genomic and transcriptomic techniques to examine underlying causes for health and biological traits in horses, livestock, exotics, and other animals.

**Dustin Yates** is an Assistant Professor of Stress Physiology in the Department of Animal Science at the University of Nebraska – Lincoln. He earned his BS degree from Texas A&M University, his MS at Angelo State University, and his PhD at New Mexico State University. He completed a postdoctoral fellowship in developmental biology with Sean Limesand at The University of Arizona. Dustin’s research program focuses on understanding the mechanisms linking prenatal stress with poor skeletal muscle growth and metabolic dysfunction.
